# A Toolbox for Isophase-Curvature Guided Computation of Metrology Hologram

**DOI:** 10.6028/jres.125.024

**Published:** 2020-08-13

**Authors:** Ulf Griesmann, Johannes A. Soons, Gufran S. Khan

**Affiliations:** 1 National Institute of Standards and Technology, Gaithersburg, MD 20899, USA; 2 Indian Institute of Technology Delhi, New Delhi 110016, India

**Keywords:** aspheric surfaces, computer-generated hologram, contour line, diffractive optics, freeform surfaces, Hilbert phase, isophase contour, lithographic layout, optical interferometry, phase function, surface metrology

**Software DOI:** https://doi.org/10.18434/M32177

## 1 Summary

We describe the algorithmic foundations of an open-source numerical toolbox, written in the Octave language [1], for the creation of computer-generated binary and multi-level holograms used in interferometric form error measurements of complex aspheric and free-form precision surfaces and wavefronts. In a typical measurement setup for this type of surface, a hologram is used to generate a test wavefront that has the design shape of the surface, which is then compared to a fabricated part using an imaging laser interferometer. The optical function of the hologram in the measurement is generally modeled with optical ray-tracing software and it can be encapsulated by a scalar optical phase function *ϕ*: ℝ^2^→ℝ The toolbox converts phase functions into equivalent binary holograms that generate the desired test wavefronts for an interferometric form error measurement. The algorithms in this toolbox take advantage of the relationship between the local properties of phase functions and the local geometry (curvature) of isophase lines. It forms the core of an efficient algorithm for the computation of optical holograms. Holograms are created in a format that can be processed by most laser- or e-beam lithography systems. While the toolbox is chiefly aimed at the creation of hologram layouts needed for measurements of precision surfaces and wavefronts, we show that the isophase-following algorithm is easily extended to phase functions with singularities and discontinuities. Such phase functions result in holograms with zone bifurcation and they can be used to generate helical wavefronts. Light beams with helical wavefronts have applications beyond surface and wavefront metrology. The toolbox also includes a family of functions for the efficient estimation and evaluation of Zernike polynomials, which are widely used in optical applications.

## 2 Software Specifications

**Table:** 

**NIST Operating Unit**	Physical Measurement Laboratory, Sensor Science Division, Surfaces and Interfaces Group
**Category**	Computer-generated holograms and diffractive optics
**Targeted Users**	Researchers in optics and metrologists in the optics industry, who need to design and fabricate optical holograms for metrology applications and other diffractive optics.
**Operating Systems**	Cross-platform
**Programming Language**	GNU Octave 4.4 and above with the Optimization and, optionally, the Parallel and Symbolic packages installed [1, 2]. The toolbox requires additional open-source toolboxes for Boolean set algebra with planar polygons, and for the creation of lithographic layout files in GDSII format [3].
**Inputs/Outputs**	The user must supply a problem-specific Octave script that defines one or more optical phase functions and associated parameters needed to characterize optical performances and physical shapes of a set of diffractive optical elements. The output is a lithographic layout of a diffractive optical element, or hologram, capable of generating the deA-sired optical phase distributions, or wavefront, when illuminated with coherent light. The layout is generated in a format suitable for submission to a fabrication facility. Layout files can be inspected with viewers or editors for electronics design layouts, such as the open-source software KLayout [4].
**Documentation**	The toolbox is accompanied by a user manual and several commented example scripts, that illustrate a range of application scenarios.
**Accessibility**	Provided by the operating system.
**Disclaimer**	https://www.nist.gov/director/licensing

## 3 Background

Traditional optical manufacturing has perfected the fabrication of imaging systems consisting of optical elements with spherical surfaces together with the development of optics design methods that sought to wrest excellent imaging performance from optical lenses and systems constructed from spherical optical elements with imperfect imaging properties [5, 6]. It has been understood since the construction of early telescopes that aspheric lens and mirror surfaces enable imaging system designs with fewer surfaces and yet far better performance, but the challenge of fabricating aspheric surfaces has tended to restrict their use to high-value applications, for example in astronomical imaging systems (telescopes), or a in certain class of earth-observing satellites. The introduction of computer-controlled fabrication systems into the manufacturing of precision optics, the availability of ever more processing power to computer-aided optical design, and the emergence of digital interferometry has catalyzed rapid advances in the design and fabrication of optical elements and imaging systems over the past three decades. Reduced fabrication costs have enabled the wide adoption of aspheric, and even free-form [7], optical elements to take advantage of better performance, reduced stray light, and compact lens sizes, of aspheric designs. Aspheric lens designs are increasingly found even in consumer optics. A striking example is the remarkable imaging performance of cameras in mobile electronic devices, which contain sophisticated aspheric imaging objectives in a volume of only about 100 mm^3^ [8].

**Fig. 1:**
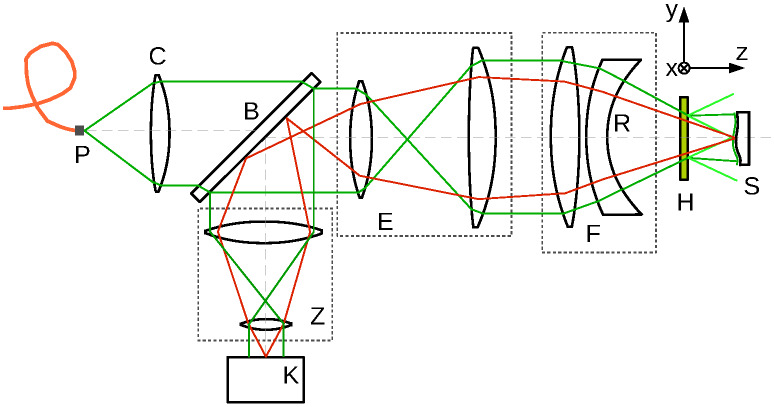
Schematic of a Fizeau interferometer configured to measure the shape error of non-spherical surfaces. Components of the interferometer setup are point source (P), collimator (C), beam divider (B), beam expander (E), Fizeau objective or transmission sphere (F), zoom objective (Z), camera (K), hologram (H), spherical reference surface (R), and non-spherical surface under test (S). Illumination rays are shown in green, imaging rays in red. (Distances and angles are not drawn to scale.)

Computer-generated holograms (CGHs) are an indispensable tool for interferometric form error measurements of ultra-precise non-spherical surfaces that require low measurement uncertainty [9-13]. CGHs can be used for surface form metrology in several different interferometer configurations. The measurement configuration sketched in Fig. 1 is common because it can be implemented easily with unmodified commercial Fizeau interferometers. In a Fizeau interferometer, coherent light from a nearly monochromatic laser is emitted by a point source, P in Fig. 1 (in practice often a slightly extended source to reduce the spatial coherence, which attenuates the deleterious effects of coherent stray light), and collimated by a lens or mirror (C). The beam is expanded by a lens pair (E), and a Fizeau objective, or transmission sphere, creates a converging beam, or sometimes a diverging beam, with a spherical wavefront (F). The hologram is placed into this test beam to generate the desired non-spherical test wavefront, typically in the first diffraction order. At the correct distance from the hologram the wavefront matches the surface of the part under test (S). All rays impinging on the test surface are normal to the surface, when the test part is free of form errors. Light reflected by the spherical reference surface of the Fizeau objective (R), and the light reflected by the test surface (S), is directed by a beam splitter towards a camera (K). An imaging system with variable magnification (Z) images the test surface onto the camera sensor. The interference fringes created by these two beams are recorded by the camera, and phase decoding techniques are used to measure their phase difference [14], which can be converted into a distance because the light wavelength is known. The measurement result is the difference between the design shape of the surface, which is encoded in the hologram, and the actual, fabricated shape of the test surface (S). The type of hologram used in this measurement setup is often called a "null-compensator" because no interferometer fringes are visible-the interferometer is "nulled"-when the surface under test has the design shape, and the test surface is correctly aligned to the hologram.

The lithographic technology needed for CGH fabrication is now widely available at research or commercial micro-fabrication foundries. However, many popular optical design software packages do not support the design of metrology holograms in lithographic layout formats (GDSII^1^1Certain commercial equipment, instruments, software, or materials are identified in this paper to foster understanding. Such identification does not imply recommendation or endorsement by the National Institute of Standards and Technology, nor does it imply that the materials or equipment identified are necessarily the best available for the purpose. [3] or OASIS [15]) that can be submitted to an open foundry. In this paper, we describe the algorithms we have developed over the past decade for the calculation of surface metrology holograms from the optical prescription of the hologram. The optical function of the hologram is calculated with optical modeling software. With the wide availability of multi-core computers, the calculation of hologram layouts is no longer the computational challenge it was merely a decade ago [16]. While our toolbox was originally developed with the goal to create improved metrology holograms at NIST, the publication of our algorithms and toolbox will enable computer-savvy optics designers and fabricators to create their own metrology holograms for precision surface form measurements. We also hope the toolbox will encourage the wider inclusion of similar algorithms and capabilities in commercial optical design software. This would make surface metrology with computer-generated holograms more widely available, and the metrology cost would be lowered.

## 4 Computer-Generated Holograms

The simplicity of an interferometer's (near-) point source illumination system, shown in Fig. 1, ensures that the hologram is illuminated with a monochromatic spherical wave of wavelength *λ* (with some spherical aberration resulting from the hologram substrate). The calculation of a hologram layout from first principles remains an enormous computational challenge and it is impractical for the large-area holograms required for metrology applications. The algorithms for hologram computation we describe here are instead based on the *Huygens-Fresnel principle* [17-21]. Despite its seeming simplicity, the Huygens-Fresnel principle enables the calculation of hologram layouts that generate test wavefronts with sufficient accuracy for form error measurements of advanced optical surfaces. The purpose of the hologram in Fig. 1 is to transform an incident spherical wave into an outgoing wave that matches the test surface after propagation through the space between hologram and test part. At the hologram, the incoming phase front must be advanced or retarded by a phase *ϕ*(**x**) (**x** is a point in the hologram plane), such that the outgoing phase front will match the shape of the test part at the correct distance. The domain of the hologram can be partitioned into half-period Fresnel zones with constant-phase boundaries that are separated in phase by *π* (equivalent to *λ /*2):

*ϕ* (**x**) = *kπ, k* ∈ℤ. (1)

The shape of the zone boundaries depends on the shape of the test part. In the simplest case, the outgoing wave is spherical and the half-period zone boundaries will be circles. When, as in the case of the Fresnel zone plate, every other Fresnel zone is made opaque, the Huygens wavelets from the remaining transparent zones will interfere constructively to generate the desired test wavefront. Alternatively, a phase difference of *π* (equivalent to *λ*/2) can be introduced in every other zone which results in a hologram that generates the test wavefront without loss of light at the opaque zones. The type of hologram that results from the Fresnel construction is called a "binary hologram" due to the alternating zone structure. It can be viewed as a generalized Fresnel zone plate [18]. The qualitative summary given here can be put on a sound mathematical foundation within the framework of scalar diffraction theory, which also clarifies the limitations of the hologram construction outlined here [22-25]. In particular, it can be shown that the width of the half-zones must be several times larger than the wavelength of light, a condition that limits the range of allowable diffraction angles and can occasionally be difficult to meet. Computer-generated holograms are generally fabricated either in the form of a patterned, opaque chromium layer on a glass substrate, which modulates the amplitude of an incoming beam such that transmitted light interferes constructively (amplitude hologram). Alternatively the hologram pattern is etched into the glass substrate to cause a phase delay of *π*, corresponding to *λ*/2, between neighboring half-zones, which enhances constructive interference (phase hologram).

The design of a metrology hologram for interferometric surface shape error measurements requires three steps. First, a phase function *ϕ*(**x**) must be found that encodes the test wavefront for a given test part. This step is typically performed using optical ray-tracing software. All optical modeling software has the option to describe the function of a CGH using an ideal, plane optical element that simply adds a phase *ϕ*(**x**) term to the optical system. In most cases, this phase function can be modeled with a suitable bivariate polynomial, for example a Zernike polynomial (see Appendix Sec. 9) or a Q-polynomial [26]. The phase function resulting from optical modeling can also compensate aberrations, for example those caused by the hologram substrate. The second step calculates the Fresnel half-zone boundaries for the phase function with the level of accuracy determined by the required form error uncertainty. Finally, the zone boundaries must be translated into a description of the zone geometry that can be processed by the fabrication system. Metrology holograms are generally fabricated with either photo-lithography or e-beam lithography. In a lithographic layout for most modern lithography systems, the Fresnel zone boundaries of a hologram must be approximated by closed polygons and stored in a layout format such as GDSII [3] or OASIS [15]. In the following sections we describe algorithms suitable for the efficient calculation of approximation polygons for phase boundary lines in Fresnel holograms *and* for the assembly of the boundary lines into closed polygons that are needed for the full description of the layout geometry.

## 5 Algorithms

At first glance, it may seem that the calculation of half-zone boundaries should not pose a problem. An efficient algorithm for the calculation of iso-surfaces was described decades ago [27], and its two-dimensional sibling, the marching squares algorithm for the calculation of iso-contours for functions *f*: (*x, y*)↦*z*, is a staple of computer science teaching [28]. Implementations of the marching squares algorithm are available for many programming languages, including Octave [*contourc*]^2^. A simple calculation, however, shows that the marching squares algorithm is not well suited for the calculation of the Fresnel half-zone boundaries defined by Eq. (1) in optics. The marching squares algorithm operates on a sampled grid of function values. For an optical hologram, the position of the polygon vertices describing Fresnel zones must have a position uncertainty well below the resolution of the lithography system. We typically use a maximum deviation of 5 nm of the polygon approximation from the isophase curve, but 10 nm would be acceptable. The spacing of isophase curves in a hologram is generally larger than the wavelength of light, usually several *µ*m. For an average-sized circular hologram with 70 mm diameter the number of required phase function evaluations is approximately 3.8 ˟ 10^13^ when the sample spacing is 10 nm. The goal of the algorithms described in the following sections is to reduce the number of required function evaluations as much as possible, because the naive application of the marching squares algorithm would result in an exorbitant number of wasted function evaluations far from the isophase curves. An additional complication is that the marching squares algorithm, at least in its common implementations, assumes the underlying function to be continuous, which is not always the case for optical phase functions.

Application specific algorithms for resource-sparing calculations of isophase curves are still required. Recently a contouring algorithm, the Pilot Approximation Trajectory (PAT) algorithm, a derivative of the marching squares algorithm, was developed for the contouring of functions with very time consuming evaluation [29]. Our algorithms similarly address the need to minimize the number of function evaluations, but unlike the PAT algorithm, they make use of the local geometry of isophase contours, which can be calculated from the local properties of the optical phase function. The algorithms are well suited to the calculation of optical holograms because phase functions are generally smooth functions that vary slowly, and derivatives can be calculated reliably. We further show in Sec. 5.5 how our geometry based algorithms can be extended to work with phase functions that have singularities and discontinuities, which occur for an important class of diffractive optics.

We begin with a brief summary of the relevant geometric relationships that are the foundation of the algorithms presented in the following sections. A curve in the plane is a function *γ: s* ↦ **x**(*s*), where *s* is the arc length commonly used to parametrize curves [30], and **x**(*s*) = (*x*(*s*), *y*(*s*)) is a point on the curve in the plane. An isophase curve has a constant phase along the curve:

*ϕ* (**x**(*s*)) = const .
(2)

Calculating the derivative of Eq. (2) with respect to the arc length parameter yields: .

$$\frac{d}{ds}\phi (x(s))=\frac{\partial \phi }{\partial x}\frac{dx}{ds}+\frac{\partial \phi }{\partial y}\frac{dy}{ds}=\nabla \phi ∙\frac{dx(s)}{ds}=0.$$

(3)

**Fig. 2:**
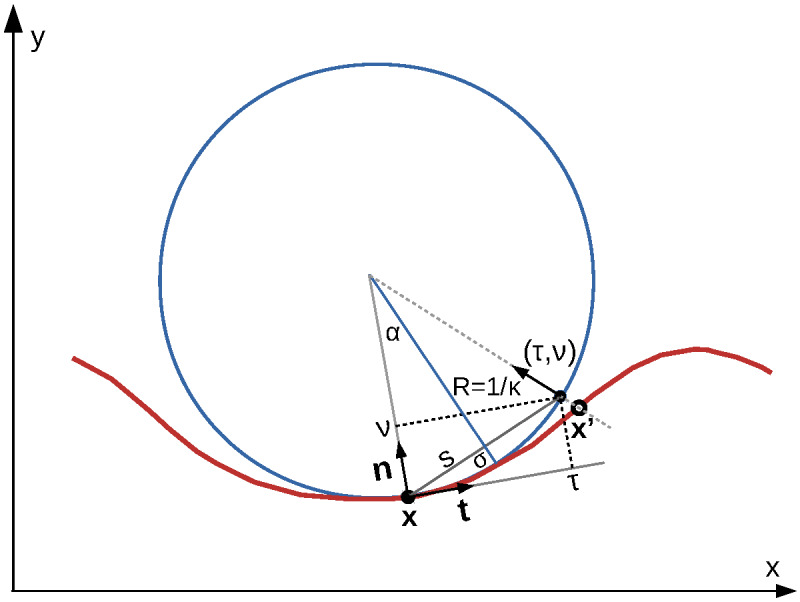
Osculating circle (blue) at a point **x** = (*x, y*) on a planar isophase curve (red). Unit tangent and normal vectors, **t** and **n**, define local coordinate frames with coordinates (*τ,v*) at each point of the isophase. At point **x** of the curve the isophase curvature *κ* is positive.

The symbol ∙ denotes the scalar (inner) product. The factor *d***x**(*s*)*/ds* in Eq. (3) is the unit tangent vector at the isophase curve when the curve is parametrized with the arc length [30]. From Eq. (3) follows the simple but very useful conclusion that the phase gradient field, ∇*ϕ* (**x**(*s*)), *is normal to isophase curves*. Equation (3) implies that a linear approximation to an isophase curve at any point on the curve can be obtained from the unit gradient vector field of the phase field *ϕ*(**x**):

$$n=\frac{\nabla \phi }{\left\|\nabla \phi \right\|}$$

(4)

because an isophase tangent vector **t** can always be found for which **t** ∙ **n** = 0. In the CGH toolbox the sign of **n** is chosen such that the exterior product **t**˄**n** *>* 0. The pair of vectors **t**(*s*) and **n**(*s*) then defines a local, right-handed coordinate system at each point of an isophase curve. A second-order approximation of an isophase curve can be obtained by considering the local curvature of the isophase. As in the case of the tangent and normal vectors, the isophase curvature *κ*(*s*) can be expressed solely through the partial derivatives of the phase function,

$$\kappa (s)=-\frac{\phi_{xx}\phi_{y}^{2}2\phi_{xy}\phi_{x}\phi_{y}+\phi_{yy}\phi_{X}^{2}}{{\left(\phi_{X}^{2}+\phi_{y}^{2}\right)}^{3/2}}$$

(5)

where the abbreviated *ϕ_x_* denotes the partial derivative *∂ϕ*(**x**(*s*))*/∂x*, etc. [*vphderiv*]. Equation (5) is central to the algorithms described in the following sections and we provide a derivation in Appendix Sec. 8. The inverse of the curvature can be interpreted as the radius *R* = 1*/κ* of a circle at a point **x**(*s*) of the isophase, the osculating circle, that shares tangent and normal vectors with the isophase curve, as shown in Fig. 2.

### 5.1 Domain Tilings

For the algorithms described here, the first step is to subdivide the hologram's domain into a set of rectangular tiles [*polytile*]. This approach has several advantages. Since the hologram in each tile area can be calculated independently of other tiles, the hologram's computation can take advantage of the multi-processor hardware that is now commonplace. The final layout of a hologram is described by a set of closed planar polygons. Subdividing the hologram domain is also an efficient way to ensure that the number of polygon vertices does not exceed the maximum number of vertices permitted in the layout files that are used by lithography tools (8192 in the case of GDSII); smaller tiles directly result in shorter polygons. Subdividing the hologram's domain can also be used to avoid exceptional points within a tile, which can result in closed or intersecting isophase curves. When an exceptional point of the phase function is detected in the interior of a tile, the tile is subdivided such that the exceptional point is located on the dividing line. Phase functions with singularities are handled similarly to ensure that phase singularities only occur on the edge of a tile. Every isophase curve then has exactly two tile edge intersections. The tiling also permits the intersections of the isophase curves with the tile edge to be ordered according to their distance from the tile origin along the edge, and isophase approximation polygons can be oriented such that their initial vertices are closer to the tile origin than their terminal vertices. Tile intersection ordering and polygon orientation is important for the isophase fill algorithms described in Sec. 5.4.

**Fig. 3:**
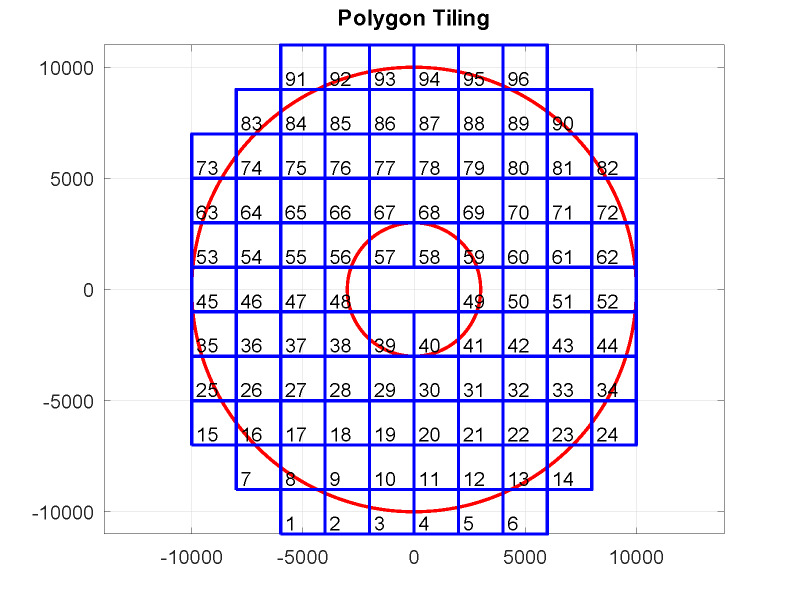
An example of a tiling with square tiles (blue lines) covering the domain of an annular hologram [*polytile*]. Two polygons (red circles) define the outer and inner boundaries of the domain. Horizontal and vertical dimensions in this example are given in µm.

Figure 3 shows the example of a tiling for a circular hologram domain with a 20 mm diameter and a central circular opening with 4 mm diameter. The width of the square tiles shown is 2 mm. The lower left corner of a tile is considered the tile origin. Each tile has four edges that circumscribe the tile in counter-clockwise direction, where adjacent edges are orthogonal. Once a hologram is calculated for all tile areas, any part of the hologram extending beyond the domain boundaries is removed ("clipped") using Boolean set algebra functions [*polybool*].

### 5.2 Pilot Approximation

Octave is an environment for numerical computing. It provides several building blocks to implement efficient algorithms for calculating optical isophase curves. We first describe an algorithm we call the pilot approximation algorithm [*phase2cgh tile pa*], that draws on the marching squares contouring algorithm [28] [*contourc*] and on an implementation of an algorithm for finding roots of continuous functions [*vfzero*] [31].

Our algorithm is similar to the Pilot Approximation Trajectory (PAT) contouring algorithm [29]. It uses the marching squares algorithm to first calculate a coarse approximation, the pilot approximation, of the isophase curves on a tile. The phase function is typically sampled on a grid with a resolution between 100 × 100 and 500 × 500 samples and the coarse contours are calculated for this grid of phase values using the built-in *contourc* function [*isophase approx*]. The vertices of the approximate isophase polygons will generally not be located on the isophase curve with the desired vertex position tolerance (typically 0.1 nm). Improved vertex positions are found by searching for the isophase at each vertex of the approximate isophase polygon in a direction normal to the polygon [*vfmatch*]. The result is a set of isophase polygons *P_k_* that have phase values Φ*_k_* where distances between the vertices and the true isophase curve are less than a predefined tolerance. Unless the curvature of the isophase curves is very low, the number of polygon vertices in the pilot approximation is not sufficient to represent the isophase without exceeding the acceptable deviation of the approximation polygon from the isophase curve, and additional vertices must be added to the polygon. The number of additional vertices required between the vertices of the pilot approximation polygon can be estimated using the osculating circle approximation of the isophase described in Sec. 5. Using Eq. (5) we calculate the isophase curvature *κ* at the midpoint between two vertices of the pilot approximation polygon. From the radius of curvature, *R* = 1*/κ*, and the allowable deviation *σ* of the isophase from the polygon segment, an allowable segment length

$$S=2\sqrt{\sigma (2R-\sigma )}$$

(6)

can be calculated by Eq. (6) (see Fig. 2). The number of additional vertices then follows from the ratio of the vertex distances in the pilot approximation polygon and the maximum allowable segment lengths.

Additional vertices are placed on the line between vertices of the approximation polygon at equal distances and the exact positions are calculated by searching for the location with the correct phase value in the direction normal to the polygon segment [*vfmatch*]. In a final step, the intersections of the isophase polygons with the tile edge are calculated [*isophase clip*]. The intersections are the initial and terminal vertices of isophase polygons traversing a tile. The steps of the pilot approximation algorithm are summarized in Alg. 1. In the implementation of the algorithm [*isophase refine*] the inner loops in Alg. 1 are replaced by vectorized expressions to speed up the computation. Once the isophase polygons over a tile are calculated, they are assembled into closed polygons using the algorithm described in Sec. 5.4.

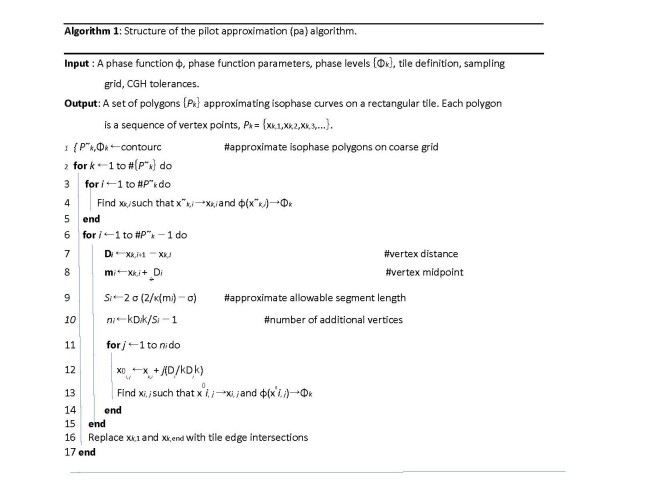



### 5.3 Isophase Following

The algorithm described in Sec. 5.2 works well for many phase functions that are encountered in precision surface metrology, but the reliance on the marching squares algorithm [*contourc*] results in limitations. One prominent example is the Hilbert phase function

$$\phi_{H}(x,p)=\arg\left[\exp\left(ip\;\arctan\left(\frac{y-y_{0}}{x-x_{0}}\right)\right)\right]$$

(7) 

which has a phase singularity at (*x*_0_, *y*_0_) and, for *p* = 1, a phase jump of 2*π* on the half-line {**x**|*x < x*_0_, *y* = *y*_0_}, when it is implemented using the *atan2* function [*phase hilbert*]. The factor *p*ϵℕ, also called "topological charge", determines the angular slope of the phase and the number of lines with discontinuous phase [32-34]. The pilot approximation algorithm described in Sec. 5.2 fails with phase 

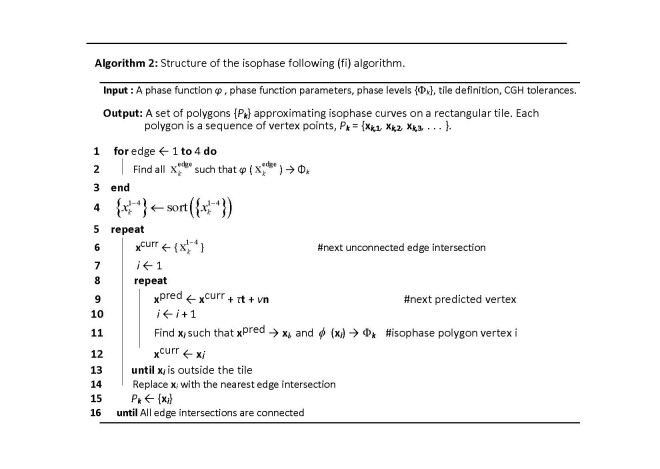



functions containing Hilbert terms shown in Eq. (7). Later in this section we describe an alternative algorithm, the isophase following algorithm, that can be extended to work with discontinuous phase functions such as those containing Hilbert phase terms.

Instead of calculating a coarse approximation of the isophase curves, the isophase following algorithm proceeds from calculating the isophase curve-tile edge intersections on all four edges of a tile [*isophase edges*]. The intersections can be ordered on the tile edge according to their distance from the tile origin (lower left tile corner) [*edge sort*]. Starting at the first tile intersection the approximate location of the first isophase polygon vertex inside the tile can be estimated using the osculating circle approximation of the isophase curve as shown in Fig. 2 [*predict xy*]. For a point on the isophase curve, the unit normal vectors **t** and **n**, and the isophase curvature *κ* = 1*/R* can be calculated using Eqs. (3) and (5). For an allowable deviation *σ* of the polygon segment from the isophase curve, the polygon segment length *S* can be calculated with Eq. (6), and the coordinates (*τ,v*) of the next polygon vertex (approximating **$x^{ʼ}$** in Fig. 2) in the **t**, **n** coordinate frame can be estimated as

$$\tau =S\sqrt{1-{\left(S/2R\right)}^{2}}$$

$$v=\frac{S^{2}}{2R}.$$

(8)

The Frenet equations [30] (also see Appendix Sec.9),

$$\frac{d\text{t}}{ds}=\kappa n\text{, and}$$

$$\frac{d\text{n}}{ds}=\mathrm{-\kappa}\text{t ,}$$

(9)

which describe the evolution of the **t**,**n** coordinate frame along the curve, can be used to estimate the isophase normal vector **n***_p_* at the predicted vertex. When the osculating circle arc length from **x** to (*τ,v*)

*L* = 2*R* arcsin

$$\left(\frac{S}{2R}\right)$$

(10) 

is used to estimate the isophase curve length in the second of the Frenet equations in Eq. (9), the normal vector **n**՛ at **x**՛ can be approximated by

**n***_p_* =
**n**-*κL***t** .

(11)

The exact vertex position **x**՛ on the isophase curve (open circle in Fig. 2) is again obtained using a level finding algorithm [*vfmatch*] in the direction of **n***_p_*. This process is repeated until the isophase polygon exits the tile area. The intersection of the last polygon segment with the tile edge is then replaced with the closest isophase-tile edge intersections initially calculated. The initial and terminal tile edge intersections are removed from the list of unconnected edge intersections. The calculation of the next isophase polygon proceeds from the next available unconnected edge intersection until all intersections are connected by isophase polygons [*isophase curves*]. A summary of the isophase following algorithm is shown in Alg. 2.

The inner loop of the isophase following algorithm, Alg. 2, is again implemented using the vector idiom of the Octave language to achieve adequate processing speed [*isophase curves*]. This need to "vectorize" the computation creates an additional problem. The isophase following procedure must proceed from only half of the isophase-tile edge intersections, and the start intersections must be chosen such that no isophase curve is traced in duplicate. The algorithm for the selection of start intersections is shown in Alg. 3 [*isophase vertex init*]. It is predicated on the assumption that tiles were designed such that all isophase curves have two tile edge intersections and isophase curves never cross. 

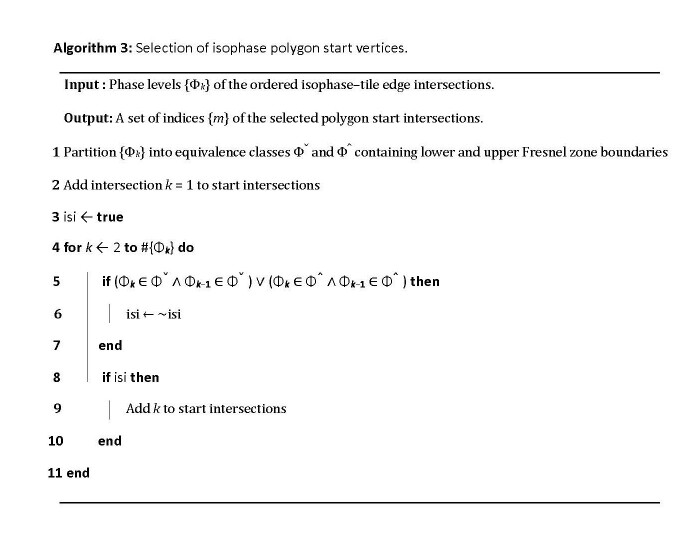



### 5.4 Isophase Filling

**Fig. 4:**
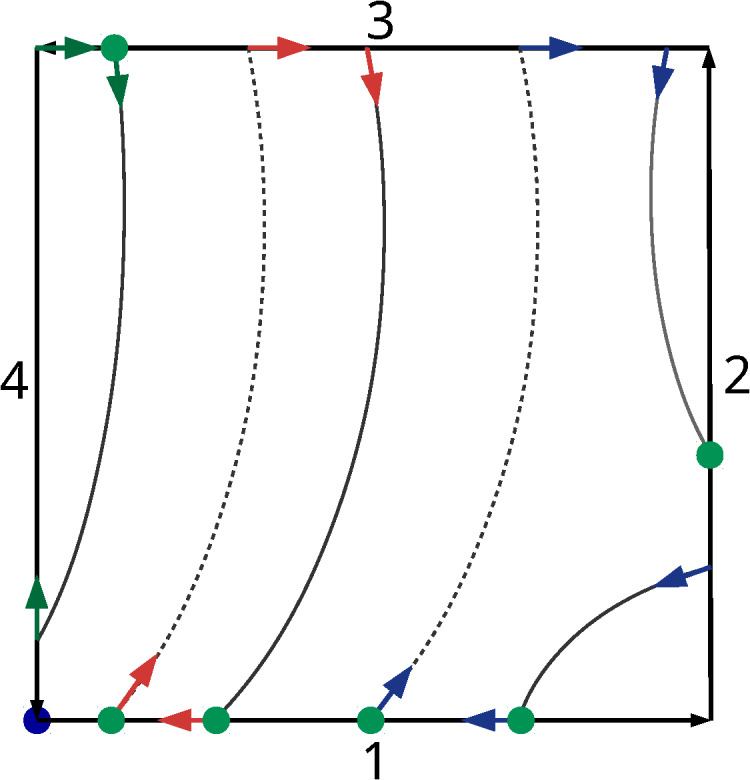
Polygons approximating isophase curves crossing a tile area. Dashed isophase curves indicate the boundary with the lowest phase value in a Fresnel zone, solid lines the boundary with the highest phase value. The first and last vertices of each polygon are tile edge crossings. Green dots indicate the location of the first (start) vertex of a polygon. The blue dot denotes the tile origin. Arrows indicate paths for the assembly of isophase or phase boundary lines into closed polygons.

The geometry of a hologram layout must be described by a set of closed polygons for most common fabrication systems that use the GDSII or OASIS [15] layout description standards. Isophase curves must be connected into closed polygons that circumscribe areas with phase values corresponding to opaque (or phase-retarded) Fresnel zones, as described in Sec. 4. An algorithm for the filling (or shading) of adjacent isophase curves [*isophase fill*] is illustrated in Fig. 4. The polygons approximating isophase curves can be oriented such that their initial vertices are closer to the tile origin than their terminal vertices as shown in Fig. 4. The algorithm picks an unconnected polygon (e.g., the polygon that is marked with red arrows and starts on edge 1) and then looks up the neighboring isophase intersections of the end vertex of the polygon in negative, **x**^-^, and positive, **x**^+^, edge direction. The phase values on the line between one of the neighbors will be in the correct phase range of the Fresnel zone, and the polygon that starts or ends on this neighboring intersection must be connected to the first polygon (see red arrows). The end of the combined polygons again has two neighbors. When the neighbor in the negative direction is the start vertex of the original polygon, a closed polygon is complete. Otherwise, another polygon needs to be connected to the already connected polygons until the condition for a closed polygon is met (see blue arrows in Fig. 4). When a polygon traverses a tile corner, it is also possible that the negative neighbor of a polygon is its own start vertex and the polygon is then connected to itself when the phase on the edge connection is in the desired phase range. This situation is illustrated with the green arrows in Fig. 4. In the CGH toolbox, the loop in Alg. 4 is implemented through recursion.

### 5.5 Discontinuous Phase Functions

The isophase following algorithm of Sec. 5.3 can be extended to phase functions with discontinuities and phase singularities. Examples are the phase functions with the Hilbert terms of Eq. (7), which will be discussed in detail in this section. Hilbert phase terms generate helical optical wavefronts characterized by an angular momentum distinct from the spin angular momentum related to polarization [34-36]. Helical beams are finding a growing number of applications in advanced imaging systems [37-39].

The isophase following algorithm relies on the derivatives of a phase function to calculate isophase normal vectors and isophase curvatures. While the derivatives exist everywhere except at the phase singularity in Eq. (7), conventional methods for numerical differentiation fail at the discontinuities. This problem can be overcome by considering the complex *continuous* function

*Z*(**x**) = *e^iϕ^*
^(^**^x^**^)^ = cos *ϕ* (**x**) +
*i* sin *ϕ* (**x**) .

(12)

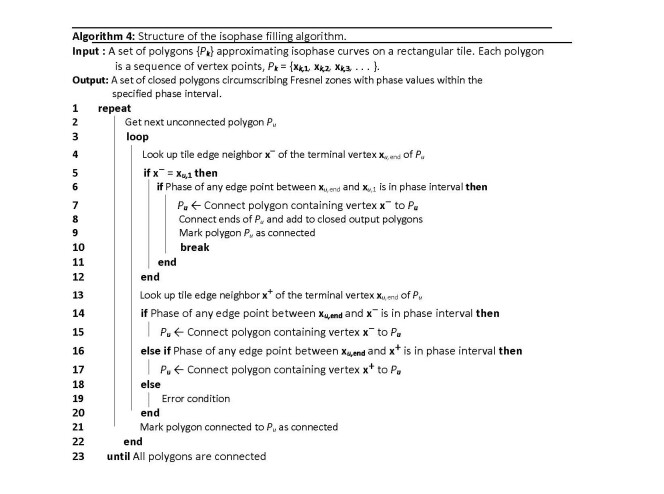



Expressions for the derivatives of discontinuous phase functions suitable for numerical differentiation can be derived by calculating the partial derivatives of *Z*(**x**) in Eq. (12).

$$\phi_{x}=-i\frac{Z_{X}}{Z}\text{, and }$$

$$\phi_{y}=-i\frac{Z_{y}}{Z}\text{, }$$

(13)

because *Z*(**x**) is continuous and its partial derivatives can be calculated at or near the discontinuities of *ϕ* (**x**) using standard methods for numerical differentiation (the abbreviated notation for partial derivatives introduced for Eq. (5) is also used in this section). The second numerical derivatives of *ϕ* (**x**) can similarly be calculated by using the derivatives of *Z*(**x**) [*vwphderiv*],

$$\phi_{xx}=-i\left(\frac{Z_{xx}}{Z}+\phi_{x}^{2}\right),\;\phi_{xy}=-i\left(\frac{Z_{xy}}{Z}+\phi_{x}\phi_{y}\right), and\;\phi_{yy}=-i\left(\frac{Z_{yy}}{Z}+\phi_{y}^{2}\right),$$

(14)

where *ϕ_x_* and *ϕ_y_* are calculated using Eq. (13). Using Eqs. (13) and (14) the isophase following algorithm in Sec. 5.3 can be adapted for the calculation of holograms with discontinuous phase functions simply by replacing the method of numerical differentiation. Only one additional change is required. Fresnel zone boundaries are no longer lines of constant phase because the phase changes by 2*π* every time a zone boundary crosses a discontinuity. The implementation of the boundary following algorithm [*boundary curves*] must test for the presence of discontinuities and adjust the phase whenever a zone boundary is crossed. The algorithm for determining the boundary polygon start vertices [*boundary vertex init*] takes into account that a phase singularity with undefined phase may be present on one of the tile edges. Similarly the boundary filling algorithm [*boundary fill*] is expanded to handle the 2*p* zone boundaries that meet at a singularity. First, boundaries are connected at the singularity, then boundary filling proceeds as usual with the algorithm described in Sec. 5.4.

## 6 Methods for Validation

The functionality of the toolbox was validated in several ways. For elementary cases, such as a rotationally symmetric Fresnel zone lens illuminated by a collimated beam, the hologram zone radii can be derived analytically [17, 19] and can be used to confirm the zone radii calculated by the toolbox from the phase function of the Fresnel lens. Holograms can be validated locally because the gradient of a phase function, ∇*ϕ* (**x**), is related to the local grating pitch *p*(**x**), the width of a Fresnel zone pair at **x**,

$$\left\|\nabla \phi (\text{x})\right\|≅\frac{2\pi }{p(\text{x})},$$

(15)

because the phase change corresponding to the zone pair is 2*π*. The relationship in Eq. (15) was used for spot checks of holograms either after the layout is calculated or after fabrication of the hologram by comparing the zone widths to the width that is expected from the phase gradient. Zone widths of finished holograms can be measured with a microscope. Finally, the toolbox was validated through the use of holograms that were made with the toolbox in a variety of interferometric tests and measurements. Some of these measurements are described in Refs. [40-45].

## 7 An Example

The user manual for the toolbox [46] describes several applications of varying complexity. Here we illustrate the toolbox with the example of a Fresnel zone lens with an additional Hilbert phase term. A Fresnel zone lens that brings a collimated beam of light with wavelength *λ* to a focus at a distance *f* from the zone lens (in first diffraction order) has the spherical phase function

$$\phi_{F}(\text{x})=\frac{4\pi }{\lambda }\left(\sqrt{f^{2}+{\left\|{\text{x-x}}_{0}\right\|}^{2}}-f\right).$$

(16)

**Fig. 5:**
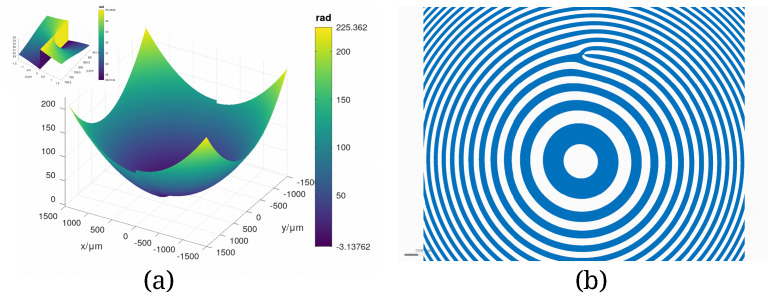
(a) Central 3 mm × 3 mm domain of a phase function consisting of a Fresnel term centered at (0 mm, 0 mm) and a Hilbert term with topological charge *p* = 2 centered at (0 mm, 0.8 mm). The inset shows a 3 μm × 3 μm patch centered at the singularity of the Hilbert term. (b) The resulting binary hologram calculated with the phase boundary following algorithm described in Sec. 5.5. The hologram shows the zone forking characteristic of phase functions with Hilbert phase terms.

**x**_0_ is the center coordinate of the phase function. In this example we place the Fresnel phase function at the coordinate origin, **x**_0_ = **0**, and assume a focal distance *f* of 100 mm and a wavelength of 0.63282 Am. The phase distribution of *ϕ_F_* + *ϕ_H_* with an added Hilbert term *ϕ_H_* in Eq. (7) with topological charge *p* = 2 at an offset (0 mm, 0.8 mm) is shown in Fig. 5(a). The binary hologram that generates this phase front when illuminated with collimated light is shown in Fig. 5(b). The boundaries between dark and light zones are lines with phase values 3*π/*4 + *kπ, k*ϵℤ. Fig. 5 illustrates the characteristic forking of Fresnel zones into *p* + 1 zones at the singularity of a Hilbert phase term.

## 8 Appendix: Isophase Curvature

Equation (5) relates the isophase curvature to the partial derivatives of a scalar phase field. In this section we give an elementary derivation of this connection, because it is central to the algorithms described in this paper. Consider a circle that tangentially touches an isophase curve at a location **x**(*s*) on the curve and intersects the curve at **x**՛(*s՛*) (also see Fig. 2). Two lines normal to the isophase curve can be defined at **x** and **x՛** ,

λ↦x+λn

μ↦x՛+μn՛,

(17)

where *λ,μ*ϵℝ. **n** is the unit normal vector at **x**, and **n՛** the unit normal vector at **x**^՛^ . The two lines intersect where

**x** + *λ***n** = **x**՛ +
*μ***n՛** .

(18)

A solution for the parameter *μ* is found when Eq. (18) is multiplied by **t**, the unit tangent vector at **x**,

$$\mu =-\frac{\text{t}\cdot (\text{x՛}-\text{x})}{\text{t}\cdot \text{n}՛}$$

(19) 

because **t**·**n** = 0. When **x**՛ is moved along the isophase curve towards **x**, the circle becomes the osculating circle of the isophase curve, shown in Fig. 2, at position **x** in the limit **x՛**→**x**. In the limit, the lines in Eq. (17) intersect at the center of the circle. Since **n** is a unit vector, *μ* must be the radius, *R*, of the circle. **x՛** and **n**^՛^ in Eq. (19) can be linearized in the vicinity of **x**. For a small arc distance *ẟs* from **x**, **x**՛ and **n՛** can be written as linear expressions in *ẟs*,

**x**՛ = **x** + **t** *ẟs* + *O*(*ẟs*^2^)

**n**՛ = **n** + $\frac{d\text{n}}{ds}$*ẟs* + *O*(*ẟs*^2^) . (20)

Replacing **x՛**and **n՛** in Eq. (19) with their linearized forms in Eqs. (20), and taking the limit *ẟs*→0, results in an equation for the curvature *K* = 1*/R* of the osculating circle (and the isophase curve) at **x**:

$$\kappa =-\frac{d\text{n}}{ds}\cdot \text{t .}$$

(21)

Equation (21) also follows from the second of the Frenet equations in Eqs. (9) when it is multiplied with **t**. The derivative of the normal vector along a curve in Eq. (21),

$$\frac{d\text{n}}{ds}=\frac{\partial \text{n}}{\partial x}\frac{dx}{ds}+\frac{\partial \text{n}}{\partial y}\frac{dy}{ds},$$

(22)

can be related to a phase function *ϕ* through the definition of the unit normal vector in Eq. (4),

$$\text{n=}\frac{\nabla \phi }{║\nabla \phi ║}=Ɲ\nabla \phi ,$$

where *Ɲ*=

$${\left(\phi_{x}^{2}+\phi_{y}^{2}\right)}^{-\frac{1}{2}}$$

(23)

For both components *n_k_* (*k* = 1, 2) of **n**, Eq. (22) has the form

$$\frac{dn_{k}}{ds}=\nabla n_{k}\cdot \frac{dx}{ds}=\nabla n_{k}\cdot t$$

(24)

(numerical indices are used to distinguish the normal vector components from derivatives). Applying ∇ to each of the components *n_k_* produces

∇*n*_1_ = ∇(*Ɲ ϕ_x_*) =
*Ɲ*∇*ϕ_x_* +
*ϕ_x_*∇*Ɲ*

∇*n*_2_ = ∇(*Ɲ ϕ_y_*) =
*Ɲ*∇*ϕ_y_* +
*ϕ_y_*∇*Ɲ,*

(25) 

which, when multiplied with **t** according to Eq. (24), and written in vectorial form again, gives

$$\left[\begin{array}{l} \nabla n_{1}\cdot t\\ \nabla n_{2}\cdot t \end{array}\right]=Ɲ\left[\begin{array}{l} \nabla \phi_{x}\cdot t\\ \nabla \phi_{y}\cdot t \end{array}\right]+(\nabla Ɲ\cdot t)\left[\begin{array}{l} \phi_{x}\\ \phi_{y} \end{array}\right]\text{.}$$

(26)

The curvature *κ* is finally obtained, when this expression for *d***n***/ds* is applied in Eq. (21):

$$\kappa =-Ɲ\left[\begin{array}{l} \nabla \phi_{X}\cdot t\\ \nabla \phi_{y}\cdot t \end{array}\right]\cdot t.$$

(27)

The second term in Eq. (26) does not contribute to the curvature because it is a vector proportional to ∇*ϕ* and thus orthogonal to **t** (Eq. 3). From the orthogonality of **t** and **n** it follows further that the unit tangent vector **t** can be written as

$$t=Ɲ\frac{\phi_{y}}{-\phi_{x}}\text{,}$$

(28)

where *Ɲ* is the same normalization factor defined in Eq. (23) for the normal vector **n**. With Eq. (28) the sought after expression for the isophase curvature *κ* in Eq. (5) is obtained

$$\begin{array}{l} \kappa =-{Ɲ}^{2}\left[\begin{array}{l} \phi_{xx}\phi_{y}-\phi_{xy}\phi_{x}\\ \phi_{xy}\phi_{y}-\phi_{yy}\phi_{x} \end{array}\right]\cdot t\\ =-{Ɲ}^{3}\left(\phi_{xx}\phi_{y}^{2}-2\phi_{xy}\phi_{x}\phi_{y}+\phi_{yy}\phi_{x}^{2}\right) \end{array}$$

$$=-\frac{\phi_{xx}\phi_{y}^{2}-2\phi_{xy}\phi_{X}\phi_{y}+\phi_{yy}\phi_{X}^{2}}{{\left(\phi_{X}^{2}+\phi_{y}^{2}\right)}^{3/2}}.$$

(29)

Interested readers can find a less pedestrian derivation by Goldman [47], that places Eq. (29) in the context of differential geometry.

## 9 Appendix: Zernike Polynomials

The CGH toolbox includes a suite of functions for the evaluation, estimation, conversion, and transformation of Zernike polynomials. Zernike polynomials are a complete set of orthogonal polynomials on a circular disk. They provide an efficient representation of arbitrary wavefront aberrations over a circular pupil. Various definitions exist for Zernike polynomials that differ in the coordinate system used, parameter numbering scheme, and normalization. Our implementation of Zernike polynomials is based on the definition described in the ANSI Z80.28-2017 standard [48, 49], which has the following elements:

⚫ The Zernike polynomials are defined in terms of polar coordinates (*ρ*,*θ*) in the XY plane of aright-handed coordinate system. The normalized radial coordinate *ρ* ranges from 0 to 1, although the implementation in the toolbox permits values *ρ >* 1. The azimuth angle *θ* is defined relative to the positive *X* -axis and increases in counter-clockwise direction. Each Zernike term is the product of three components: a normalization factor, a polynomial defined on the radius *ρ*, and a sinusoid defined on the azimuth angle *θ*.
⚫ Each Zernike term, except the offset (piston) term, is normalized so that its variance equals 1 on the unit disk. The Zernike terms are orthonormal.
⚫ Each Zernike term is identified by two indices *n* and *m*. The radial order *n* is the degree (highest power) of the radial polynomial. The angular order *m* is the azimuthal frequency of the angular term. The latter term equals sin(|*m*|*θ*) for *m <* 0 and cos(*mθ*) for *m >*= 0. For each radial order *n*, the valid values for *m* equal -*n,* -*n* + 2, ···, *n*-2, *n*. A Zernike term can also be identified by a single index *j* = (*n*(*n* + 2) + *m*)*/*2.

Following this convention, a Zernike polynomial with coefficients $a_{n}^{m}$ is defined as:

$$Z(p,\theta )=\sum_{n=0}^{k}\sum_{n=-n}^{m}a_{n}^{m}Z_{n}^{m}(\rho ,\theta ),$$

(30)

with Zernike terms

$$Z_{n}^{m}(\rho ,\theta )=N_{n}^{m}R_{n}^{m}(\rho )\left\{\begin{array}{l} \sin\left(\left|m\right|\theta \right)\text{ if }m<0\\ \cos(m\theta )\text{ if m}⩾\text{0 .} \end{array}\right.$$

(31)

The radial polynomial term *$R_{n}^{m}$*(*ρ*) is given by:

$$R_{n}^{m}(\rho )=\left\{\begin{array}{l} \sum_{k=0}^{(n-\left|m\right|)/2}\frac{{\left(-1\right)}^{k}(n-k)!}{k!\left(\frac{n+\left|m\right|}{2}-k\right)!\left(\frac{n+\left|m\right|}{2}-k\right)!}\rho^{n-2k}\text{ if }n-\left|m\right|\text{ is even}\\ \;0\text{ if }n-\left|m\right|\text{ is odd} \end{array}\right.$$

(32)

and the normalization factor,

$$N_{n}^{m}=\sqrt{\frac{2(n+1)}{1+\delta_{m}}},$$

(33)

where *δ_m_* is 1 for *m* = 0, and 0 otherwise. The first fifteen Zernike polynomial terms are summarized in Tables 1 and 2. 

**Table 1:** Zernike indices *j* for the first fifteen Zernike terms according to the ANSI, Optical Shop Testing 2nd edition (OST), Noll, and Fringe or University of Arizona (UoA) term ordering conventions [*zern index trans*]. The symbol (-) indicates that the respective Zernike term is negated. The normalized terms *$Z_{n}^{m}$* in column 7 and their names are those found in the ANSI standard. Note that the OST and Fringe Zernike terms are not normalized.

*n *	*m *	ANSI [48]	OST [50]		Noll [51]	Fringe [52]	*$Z_{n}^{m}$*, see Eq. (31)	Common name [48]
0	0	0		1	1	0	1	piston
1	-1	1		3	3	2	2*ρ* sin(θ)	vertical tilt
1	1	2		2	2	1	2*ρ* cos(*θ*)	Horizontal tilt
2	-2	3		4	5	5	√6*ρ*^2^ sin(2*θ*)	oblique astigmatism
2	0	4		5	4	3	√3(2*ρ*^2^-1)	defocus (power)
2	2	5		(-)6	6	4	√6*ρ*^2^ cos(2*θ*)	astigmatism
3	-3	6		(-)10	9	10	√8*ρ*^3^ sin(3*θ*)	oblique trefoil
3	-1	7		9	7	7	√8(3*ρ*^3^-2*ρ*)sin(*θ*)	vertical coma
3	1	8		8	8	6	√8(3*ρ*^3^-2*ρ*)cos(*θ*)	horizontal coma
3	3	9		(-)7	10	9	√8*ρ*^3^cos(3*θ*)	horizontal trefoil
4	-4	10		(-)11	15	17	√10*ρ*^4^sin(4*θ*)	
4	-2	11		12	13	12	√10(4*ρ*^4^-3*ρ*^2^)sin(2*θ*)	secondary oblique ast.
4	0	12		13	11	8	√5(6*ρ*^4^-6*ρ*^2^+1)	primary spherical
4	2	13		(-)14	12	11	√10(4*ρ*^4^-3*ρ*^2^)cos(2*θ*)	secondary astigmatism
4	4	14		15	14	16	√10*ρ*^4^cos(4*θ*)	

**Table 2:** The first sixteen Zernike terms $Z_{n}^{m}$ and their index values *j* according to ANSI, Optical Shop Testing 2nd edition (OST), Noll, and Fringe (UoA) term ordering conventions. Note that the OST and Fringe (UoA) Zernike terms are not normalized.

j	ANSI [48]	OST [50]	Noll [51]	Fringe [52]
0	$Z_{0}^{0}$			$Z_{0}^{0}$
1	$Z_{1}^{-1}$	$Z_{0}^{0}$	$Z_{0}^{0}$	$Z_{1}^{1}$
2	$Z_{1}^{1}$	$Z_{1}^{1}$	$Z_{1}^{1}$	$Z_{1}^{-1}$
3	$Z_{2}^{-2}$	$Z_{1}^{-1}$	$Z_{1}^{-1}$	$Z_{2}^{0}$
4	$Z_{2}^{0}$	$Z_{2}^{-2}$	$Z_{2}^{0}$	$Z_{2}^{2}$
5	$Z_{2}^{2}$	$Z_{2}^{0}$	$Z_{2}^{-2}$	$Z_{2}^{-2}$
6	$Z_{3}^{-3}$	$-Z_{2}^{2}$	$Z_{2}^{2}$	$Z_{3}^{1}$
7	$Z_{3}^{-1}$	$-Z_{3}^{3}$	$Z_{3}^{-1}$	$Z_{3}^{-1}$
8	$Z_{3}^{1}$	$Z_{3}^{1}$	$Z_{3}^{1}$	$Z_{4}^{0}$
9	$Z_{3}^{3}$	$Z_{3}^{-1}$	$Z_{3}^{-3}$	$Z_{3}^{3}$
10	$Z_{4}^{-4}$	$-Z_{3}^{-3}$	$Z_{3}^{3}$	$Z_{3}^{-3}$
11	$Z_{4}^{-2}$	$-Z_{4}^{-4}$	$Z_{4}^{0}$	$Z_{4}^{2}$
12	$Z_{4}^{0}$	$Z_{4}^{-2}$	$Z_{4}^{2}$	$Z_{4}^{-2}$
13	$Z_{4}^{2}$	$Z_{4}^{0}$	$Z_{4}^{-2}$	$Z_{5}^{1}$
14	$Z_{4}^{4}$	$-Z_{4}^{2}$	$Z_{4}^{4}$	$Z_{5}^{-1}$
15	$Z_{5}^{-5}$	$Z_{4}^{4}$	$Z_{4}^{-4}$	$Z_{6}^{0}$

The Zernike polynomial related functions of the CGH toolbox can be grouped into the following categories:

⚫ **Evaluation** of a Zernike polynomial [*zern eval*]. In our implementation, we use the modified Kintner method [53, 54] for the fast and stable evaluation of the radial polynomial terms *$R_{n}^{m}$*(*ρ*) using a recursion relation. Zernike terms can be calculated and evaluated in their symbolic form in both polar and cartesian coordinates [*zern symbolic*]. This functionality requires installation of Octave's Symbolic package [2].
⚫ **Estimation** of a Zernike coefficient vector from wavefront aberration data [*zern estim*]. The estimation minimizes the least squares error and can be performed with or without the assumption of orthogonality.
⚫ **Conversion** of a Zernike coefficient vector from one definition to another [*zern index trans*]. The implemented definitions are
  - (*n, m*) index pairs defined in the ANSI Z80.28-2017 standard.
  - Zernike term index *j* defined in the ANSI Z80.28-2017 standard. Starting with an index value of 0, a polynomial with a lower value of *n* is ordered first. For equal values of *n*, a polynomial with a lower value of *m* is ordered first.
  - Zernike indices and Zernike polynomials defined in the second edition of Optical Shop Testing [50]. This publication uses a similar index numbering approach to the ANSI Z80.28-2017 standard. However, the Zernike terms are not normalized, i.e., their values on the unit circle are in the interval [-1, 1]. The lowest index value for *j* is 1. Furthermore, the azimuth angle lθʹ is defined relative to the positive *Y* -axis and increases in the clockwise direction. The sinusoidal term for an angular order l is sin(lθʹ) for l *>* 0 and cos(lθʹ) for *l<*= 0. Finally, for the same radial order *n*, cos terms have a lower Zernike index number than sin terms.
  - indices and Zernike polynomials defined by Noll [51] and also described in the third edition of Optical Shop Testing [55]. Here polynomials are defined using the same normalization and azimuth angle as the ANSI Z80.28-2017 standard. However, for a given *$Z_{n}^{m}$* term, the equivalent index *j* is defined in a different manner. Starting with *j* = 1, a polynomial with a lower value of *n* is ordered first. For equal values of *n*, a polynomial with a lower value of the azimuthal frequency |*m*| is ordered first. An even *j* corresponds to a cos(*mθ*) angular term whereas an odd *j* corresponds to a sin(|*m*|*θ*) angular term.
  - "Fringe" indices and Zernike polynomials as defined by Loomis [52, 56]. Here the azimuth angle definition is the same as that of the ANSI Z80.28-2017 standard. The Zernike terms are not normalized. This term ordering scheme is inspired by the needs of optics fabricators and groups terms according to their *n* + |*m*| values. Thus, the first group *n* + |*m*| = 2 contains the paraxial wavefront properties. The second group *n* + |*m*| = 4 contains the third order aberrations. The third group *n* + |*m*| = 6 contains the fifth order aberrations, etc. Starting with *j* = 0, a polynomial with a lower value of *n* + |*m*| is ordered first. For polynomials with the same *n* + |*m*| value, a polynomial with a higher value of the azimuthal frequency |*m*| is ordered first. Finally, cos(*mθ*) terms are ordered before sin(|*m*|*θ*). The toolbox also contains a conversion for Zernike coefficient vectors that follow this definition, but where the angle is defined relative to the positive *Y* -axis in clockwise direction.
⚫ **Transformation** of a Zernike coefficient vector to describe a mirror operation, scaling, rotation, or translation [57] [*zern transform*]. These functions can also be used to obtain the Zernike coefficient vector of a circular sub-aperture.
